# Structure of a Fe_4_O_6_-Heteraadamantane-Type Hexacation Stabilized by Chelating Organophosphine Oxide Ligands

**DOI:** 10.3390/ma14226840

**Published:** 2021-11-12

**Authors:** Anna Pietrzak, Jannick Guschlbauer, Piotr Kaszyński

**Affiliations:** 1 Institute of General and Ecological Chemistry, Łódź University of Technology, Żeromskiego 116, 90-924 Łódź, Poland; 2 Centre of Molecular and Macromolecular Studies, Polish Academy of Sciences, Sienkiewicza 112, 90-001 Łódź, Poland; J.Guschlbauer@gmx.de (J.G.); piotrk@cbmm.lodz.pl (P.K.); 3 Department of Chemistry, Middle Tennessee State University, Murfreesboro, TN 37132, USA; 4 Faculty of Chemistry, University of Łódź, Tamka 12, 91-403 Łódź, Poland

**Keywords:** heteraadmantane, organophosphine oxide ligand, single-crystal, Hirshfeld surface analysis, enrichment ratio

## Abstract

Metal-containing heteraadamantanes are compounds of interest due to their spectroscopic and magnetic properties, which make them promising materials for non-linear optics and semiconductors. Herein we report the comprehensive structural characterization of a new coordination compound of the formula [(*µ*-OH′)_2_(*µ-*OH″)_4_(O = P(Ph_2_)CH_2_CH_2_(Ph_2_)P = O)_4_{Fe(*t*-BuOH)}_4_](PF_6_)_4_(Cl)_2_ with the chelating ligand Ph_2_P(O)-CH_2_CH_2_-P(O)Ph_2_. The compound crystallizes as a polynuclear metal complex with the adamantane-like core [Fe_4_O_6_] in the space group *I*-43d of a cubic system. The single-crystal XRD analysis showed that the crystal contains one symmetrically independent octahedrally coordinated Fe atom in the oxidation state +3. The adamantine-like scaffold of the Fe complex is formed by hydroxy bridging oxygen atoms only. Hirshfeld surface analysis of the bridging oxygen atoms revealed two types of *µ*-OH groups, which differ in the degree of exposure and participation in long-range interactions. Additionally, the Hirshfeld surface analysis supported by the enrichment ratio calculations demonstrated the high propensity of the title complex to form C-H^…^Cl, C-H^…^F and C-H^…^O interactions.

## 1. Introduction

Heteraadamantanes are compounds in which some or all carbon atoms in the adamantane (tricyclo[3.3.1.1^3,7^]decane) skeleton are replaced with non-carbon atoms (heteroatoms). The heteraadamantane structural motif is commonly observed in solids, e.g., of the chalcopyrite-type, the sphalerite-type or the cristobalite-type. Less common, nevertheless still well-known, are inorganic and organometallic molecules with one discrete, zero-dimensional [M_4_E_6_] (or [M_6_E_4_]) molecular unit. As polynuclear metal complexes with unique geometrical properties, molecular heteraadamantane compounds have gained recent interest due to two main reasons: (i) they may act as potential precursors to metal chalcogenites, metal oxides, and alloys [1], and (ii) their spectroscopic and magnetic properties make them promising materials for non-linear optics [2] and semiconductors [3]. Of particular interest is the latter class of heteraadamantanes containing paramagnetic transition metal ions, such as Fe(III). Moreover, mixed oxo/hydroxo adamantane-like Fe complexes are of interest as potential models for ferritin, an iron storage protein [4]. From the crystal engineering point of view, adamantane-like cages may serve as basic building blocks for the formation of zeolite-type supramolecular networks [5].

The structural motif of the Fe/O adamantane-type cage is known with anionic and neutral polydentate ligands [4,6,7]. It has, however, never been observed with a chelating Ph_2_P(O)-CH_2_CH_2_-P(O)Ph_2_ ligand, which might be of interest due to potential anisotropic magnetic properties of such clusters.

Herein we report the structure of [(*µ*-OH′)_2_(*µ*-OH″)_4_(*µ*-L)_4_{Fe(*t*-BuOH)}_4_](PF_6_)_2_(Cl)_4_ (**I**) that contains the [Fe_4_O_6_] heteraadamantane framework stabilized by solvent molecules, in which the Fe(III) centers are octahedrally coordinated and chelated by phosphine oxide ligands. We analyzed the Hirshfeld surfaces (HSs) of bridging oxygen atoms *µ*-OH′ and *µ*-OH″ to explore the intramolecular effects characterizing the adamantane-like core. Moreover, for the supramolecular features characterization of the cation of **I**, HS and relevant fingerprint (FP) maps were generated. The analysis, supported by the enrichment ratio calculation, enabled the determination of the propensity of structure **I** to the formation of particular intermolecular interactions.

## 2. Methods

### 2.1. Synthesis

Following the reported procedure [8], a mixture of [*closo*-1,12-C_2_B_11_H_10_(CN)_2_] (0.011 g, 0.06 mmol, 1.0 eq.), [(Cp)(dppe)FeCl] (0.063 g, 0.11 mmol, 2.1 eq.) and NH_4_PF_6_ (0.027 g, 0.17 mmol, 3.0 eq.) was refluxed in dry THF (10 mL) for 18 h protected from air in a standard way (Ar-filled balloon attached via septum). The emerged orange suspension was filtered and 20 mg of this product was diluted in a mixture of CH_2_Cl_2_ and 1,2-dichloroethane. This solution was layered with a mixture of petroleum ether, Et_2_O, and THF in a closed but not air-tight glass vessel at room temperature (unprotected from sun radiation). A drop of etanol containing a few percent of *t*-BuOH was added to aid crystallization. After three weeks, several transparent, dark orange, block-shaped single-crystals emerged (<3 mg), and their structure was determined by single-crystal XRD analysis to reveal that of compound **I**. The remaining solution was evaporated, and the resulting material was purified by precipitation from CH_2_Cl_2_ solution with petroleum ether giving the intended [{(Cp)(dppe)Fe}_2_(*closo*-1,12-C_2_B_10_H_10_-1,12-(CN)_2_)](PF_6_)_2_ in 58% yield as an orange polycrystalline powder [8].

#### Characterization of Compound **I**

FT-IR (Fourier-transform infrared spectroscopy) spectrum of a solid sample of **I** in a KBr pellet (1:50 ratio) is consistent with the structure. It revealed a broad cluster of signals characteristic for the O–H groups in the range of *v* 3200–3650 cm^−1^. The intense signals observed at *v* 1167 and 1135 cm^−1^ can be ascribed to the stretching vibrations of the P = O group. Both are modestly shifted from the positions found for the free ligand (*v* 1183 and 1128 cm^−1^) due to coordination to a metal center [9,10]. Other significant signals in the IR spectrum of **I** are found at *v* 1097 cm^−1^, presumably due to the presence of the C–O bond, and at *v* 842 cm^−1^ ascribed to the stretching vibration of the P–F bond (*v* 838 cm^−1^ reported for LiPF_6_) [11].

Mass spectrometry of **I** dissolved in MeCN performed by the TOF-ES(+) technic showed the [M + H]^+^ peak of Ph_2_P(O)CH_2_CH_2_P(O)Ph_2_ as the base peak (*m/z* calcd 431.1330, found 431.1308) and signals at 524 (51%), 946 (40%), 1419 (23%). Signal at 584 (18%) could be attributed to [(Ph_2_P(O)CH_2_CH_2_P(O)Ph_2_)_4_Fe_4_(OH)_6_(PF_6_)_2_]^4+^ (*m/z* calcd 584.0463, found 584.0175). Copies of the spectra are shown in the Supplementary Materials.

### 2.2. Crystallography

A suitable orange, block-shaped single crystal of I with dimensions 0.41 × 0.27 × 0.17 mm^3^ was selected and mounted on a XtaLAB Synergy, Dualflex, Pilatus 300K diffractometer (Rigaku Corporation, Tokyo, Japan). The crystal was kept at T = 278(2) K during data collection. The structure was solved with the ShelXT 2018/2 [12] solution program (Version 2018/2, 2018, Göttingen, Germany) using Intrinsinc Phasing method and by using Olex2 1.5 (Version: 1.5, 2021, OlexSys, Durham, UK) [13] as the graphical interface. The model was refined with ShelXL 2018/3 [14] using full matrix least-squares minimization on *F*^2^. The details of crystal data and structure refinement factors are included in Table 1. CCDC 2,109,754 contains crystallographic data.

### 2.3. Hirshfeld Surface Analysis

The CrystalExplorer21 software (Version: 21.5, 2021, University of Western Australia, Nedlands 6009, Australia) was applied to generate Hirshfeld surfaces (HS) of respective structural fragments. The normalized contact distance *d_norm_* was used to visualize relevant contacts in the crystal packing. The applied molecular geometry was derived from the crystal structure. Relevant distances from the HS to the nearest atom (*d_i_*) and the exterior to the surface (*d_e_*) were plotted as scattergrams, namely fingerprints (FP) [15,16,17,18]. Moreover, the enrichment ratios (ER) for meaningful contacts between atom pairs were calculated [19].

## 3. Results and Discussion

An attempted preparation of a dinuclear complex [{(Cp)(dppe)Fe}_2_(*closo*-1,12-C_2_B_10_H_10_-1,12-(CN)_2_)](PF_6_)_2_ [8] gave minor quantities of transparent, orange, single crystals suitable for a single crystal X-ray measurement. The refined structure did not match the intended complex. Instead, analysis revealed [(*µ*-OH’)_2_(*µ*-OH’’)_4_(*µ*-L)_4_{Fe(*t*-BuOH)}_4_](PF_6_)_2_(Cl)_4_, a hexacationic polynuclear complex **I** with the [Fe_4_O_6_] heteraadamantane framework. The formation of **I** was surprising, since it required the presence of phosphine oxide Ph_2_P(O)-CH_2_CH_2_-P(O)Ph_2_ (L) and Fe(III) ions. Both species were apparently generated *in situ* by decomposition of the staring ferrocene complex (Cp)(dppe)FeCl and aerial oxidation of both the diphosphine ligand dppe and the Fe(II) ion. The components necessary to the formation of **I** could also be formed by the decomposition of the [(Cp)(dppe)Fe]^+^ fragment in the intended dinuclear complex. The CN–Fe coordination bond in the dinuclear complex of [*closo*-1,12-C_2_B_10_H_10_-1,12-(CN)_2_] has a relatively low stability in comparison to those of other dinitriles NC–X–CN, for which the formation of byproduct **I** was not observed [8].

### 3.1. Crystal Structure

**Figure 1:**
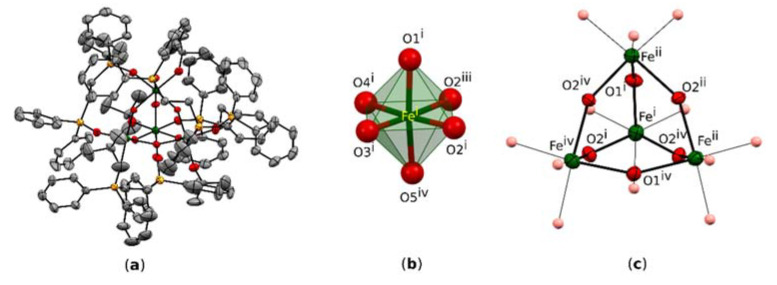
(**a**) The molecular structure of **I** and the core cation. Displacement ellipsoids are drawn at the 30% probability level; (**b**) the iron coordinate polyhedron; (**c**) view of the adamantane-like geometry exhibited by the [Fe_4_O_6_] core in **I**; symmetry codes: ^(i)^ x, y, z; ^(ii)^ x, 1−y, ½ −z;^(iii)^ 1 ¼−x, ¾−y, ¼ +z; ^(iv)^ 1 ¼−x, −¼ +y, ¾−z; hydrogen atoms are omitted for clarity.

Compound **I** crystallizes in the space group *I*-43d of a cubic system with one symmetrically independent Fe atom (Figure 1a). The Fe atom is in the oxidation state +3 and is octahedrally coordinated by six O-atoms: three O-atoms of the heteraadamantane framework (O1 and 2×O2), two O-atoms from two symmetrically independent phosphine oxide ligands L (O4 and O5), and one O-atom (O3) of a coordinating solvent molecule, *t*-BuOH (Figure 1b). Both oxygen atoms O2 of the [Fe_4_O_6_] core are placed on a 4-fold rotoinversion axis and the resulting molecular symmetry of the cation is *S*_4_. One L is bound to two neighboring Fe centers (Figure 1c). The bridging O-atoms of the heteraadamantane framework consist of six [*µ*-OH]^–^ units.

**Figure 2:**
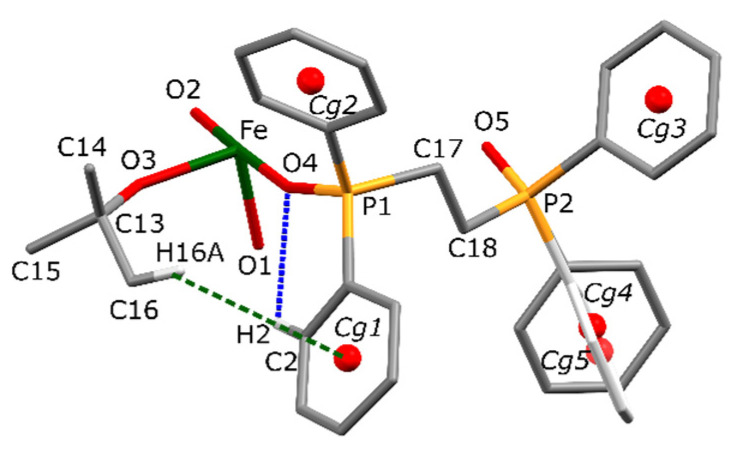
Selected short intramolecular interactions stabilizing the conformation of the cation of **I**. CH^…^O type hydrogen bond (blue dotted line) and CH^…^π interactions (green dotted lines). Hydrogen atoms not involved in particular interactions are omitted for clarity. *Cg*n represents centroids of phenyl rings. *Cg*4 and *Cg*5 refer to two sites of the disordered phenyl fragment. Only a symmetrically independent part of the molecule is presented.

The conformation of the cation is stabilized by an extensive network of short contacts. Steric effects are represented by many homonuclear C-H^…^H-C interactions. However, these are supported by weak electrostatic effects due to C-H^…^O and C-H^…^π bondings (Figure 2). The geometrical parameters of these interactions are summarized in Table 2.

**Figure 3:**
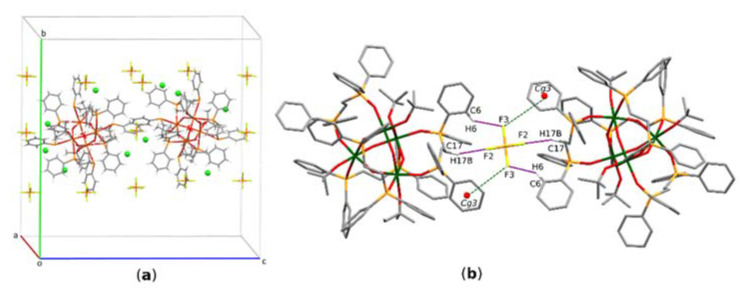
(**a**) Partial molecular packing of **I**; (**b**) C-H^…^F and F^…^π interactions in **I**. Green spheres symbolize approximated Cl^−^ ions.

In the crystal, each cation of complex **I** is surrounded by PF_6_^−^ and Cl^−^ ions (Figure 3a). The structure is stabilized by an extensive network of C-H^…^π interactions of the linking phenyl rings of the neighboring molecules. However, the crystal packing results mostly from the electrostatically driven C-H^…^F and F^…^π interactions [20] between the phenyl rings of the ligand L and fluorine atoms of the PF_6_^−^ anion. Thus, each PF_6_^−^ anion links two neighboring complexes (Figure 3b). The geometrical parameters characterizing these interactions are presented in Table 3. The H^…^F distance of 2.46(1) Å, defining the C17-H17B^…^F2 hydrogen bond, is shorter than the mean H^…^F distance of 2.53 Å reported for the C-H^…^F (PF_6_^−^) interactions, while the C6-H6^…^F3 distance is longer than the mean value. Similarly, the respective D-H^…^A angle values indicate that the C17-H17B^…^F2 hydrogen bond (145˚) is stronger than that in C6-H6^…^F3 (124˚). The former value is close to the mean angle of 143° reported in the literature for such interactions [21]. The F^…^π interaction between the PF_6_^−^ moiety and the phenyl ring *Cg*3 is defined by two values, i.e., the distance between fluorine atom and the centroid of the ring (4.057 Å), and the distance between fluorine atom and the respective atom-to-plane distance (3.165 Å). These values are also within the mean values reported in the literature for similar interactions.

**Figure 4:**
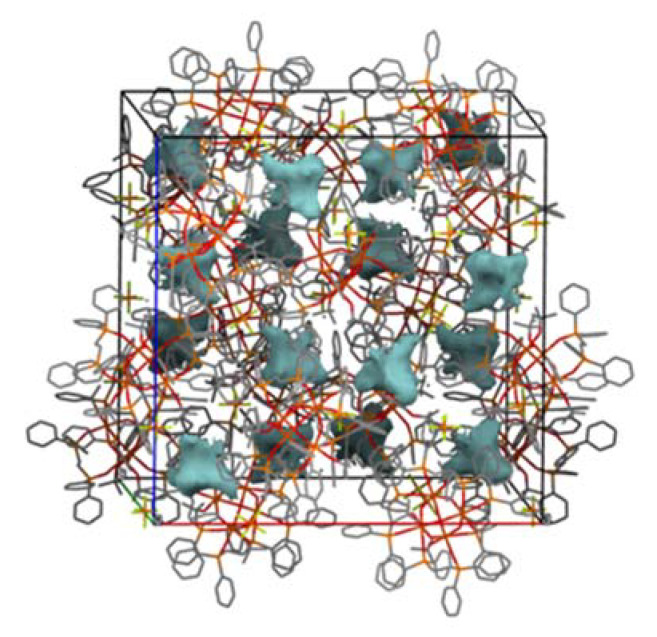
Crystal packing of **I** with voids (solid shapes) containing disordered Cl^−^ ions. Hydrogen atoms are omitted for clarity.

The structure is stabilized by interactions regarded as weak. However, the crystal structure contains quite large solvent-accessible voids, which constitute 16,3% of the unit cell volume (Figure 4).

Calculated voids are intersected by 3-fold rotoinversion axes and their total volume per asymmetric unit is *ca.* 100 Å. Solvent mask analysis, as implemented in Olex software (Version: 1.5, 2021, OlexSys, Durham, England) [11], revealed that part of the void is filled with 18 electrons. This is consistent with the presence of one Cl^−^ ion per asymmetric unit resulting in four Cl^−^ ions per formula unit. For visualization purposes, the anions are placed symmetrically around these special positions. Therefore, one Cl^−^ ion (Cl4) is not fully occupied (occupancy 0.33). Although these approximated positions do not reflect the exact geometry of their intermolecular interactions, it is obvious that these electrostatic interactions strongly support the C-H^…^F interactions in the stabilization of the crystal lattice of **I**.

### 3.2. CSD Review and Geometrical Analysis

**Scheme 1:**
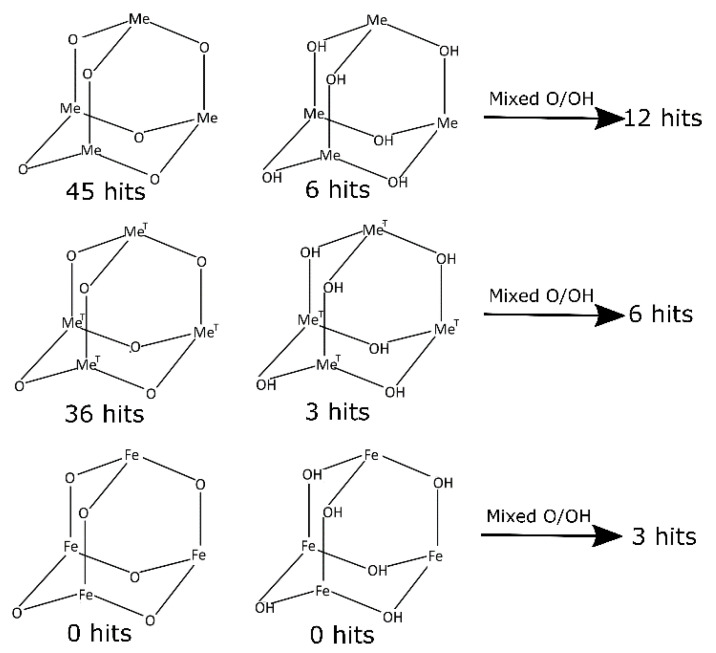
Structural models used for CSD searches. Notation: Me—any metal, O—oxo and OH-hydroxo bridging atoms, Me^T^-any transition metal.

**Figure 5:**
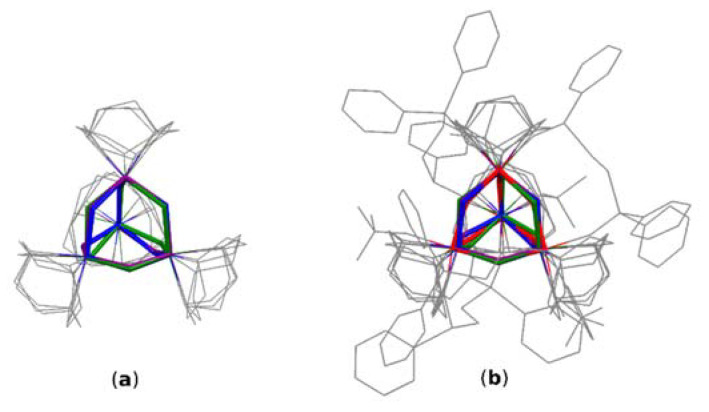
(**a**) The overlay of the [Fe_4_O_6_] cores of selected structures from CSD (**b**) The overlay of [Fe_4_O_6_] cores of selected structures from CSD and structure **I**. Only compared cores are colored and hydrogens are omitted for clarity. **I**—red, EYAVIJ—purple, JAZZUF—blue, YAXZON—green.

For a better insight into the geometrical analysis of **I**, data obtained from a depth CSD (Cambridge Structural Database) search (Scheme 1) have been reviewed (CSD version 5.42, November 2020). To get relevant data for comparative studies, the search criteria were limited to only oxo and hydroxo bridged structures containing adamantane-like moieties. Structures containing other oxygen-carrying bridges (for example alkoxy and carboxy bridges) were excluded from the search. Similarly, structures containing polyadamantane-like and other convoluted cores were omitted. The first search performed for single-crystal structures containing any metal atom (Me) at the bridgehead positions of the heteraadamantane skeletone revealed 45 oxo-bridged, 6 hydroxo-bridged, and 12 mixed oxo-/hydroxo- bridged structures. Details of the search results for mixed oxo-/hydroxo bridged structures are summarized in Table S8 (see the Supplementary Materials). Then, narrowing down the search criteria to structures containing transition metals (Me^T^) at the bridgehead positions resulted in 36 structures with oxo bridging oxygen atoms, 3 hydroxo, and 6 with mixed bridges. From the point of view of relevance to structure **I**, the most interesting are results concerning structures with iron atoms at the bridgehead positions, which could serve as models for a comparison of geometrical parameters characterizing the [Fe_4_O_6_] heteraadamantane cage. However, the CSD does not contain any relevant structure with purely oxo or hydroxo bridged cages. Instead, there are three [Fe_4_O_6_] structures with mixed bridges, namely EYAVIJ, JAZZUF, and YAXZON [4,22,23]. Interestingly, all of these complexes are chelated by triazacyclononane ligand (Figure 5a). On the other hand, there are 120 depositions containing the [Fe_4_O_6_] fragment, which include, however, structures with polyadamantane-like skeletons and other more complex systems involving oxygen atoms from organic and/or organometallic ligands. Interestingly, CSD contains only two structures of Fe complexes chelated by the Ph_2_P(O)-CH_2_CH_2_-P(O)Ph_2_ ligand [24,25], although no structure with [Me_4_O_6_] heteraadamantane core stabilized by this ligand is known thus far.

It needs to be mentioned that the results obtained from the CSD search should be carefully verified, because it is often hard to identify all hydrogen atoms by X-ray analysis. Especially, hydrogen atoms placed in the vicinity of heavy atoms may be doubtful, and many deposited structures are affected by this uncertainty. For example, the structure assigned to the JAZZUF refcode is depicted in the 2D search diagram as containing two oxo (O_oxo_) and four hydroxo (O_OH_) bridging oxygen atoms, while in the 3D structure visualizer all bridging atoms are identified as oxo bridges. Bond length analysis confirms however, the correctness of the 2D diagram.

Types of bridging oxygen atoms can be distinguished through a comparison of their respective bonding characteristics [26]. According to the International Tables for Crystallography, a typical mean bond length for Fe-O_oxo_ is 1.794 Å, while the mean Fe-O bond length in the reported structure is 1.961 Å and is consistent with the typical value reported for the Fe-O_OH_ distance (1.967 Å). A comparison of the cage parameters of structurally similar systems (Table 4) confirmed the hydroxy character of the bridging oxygen atom in **I**. Due to various symmetries governing the cage geometry of selected structures, one parameter may be defined by more than a single value. Therefore, for comparison purposes relevant mean values and their ranges (min/max) have been used (Table 4). A comparison of Fe-O bond lengths of similar systems confirmed that bridging oxygen atoms in **I** are hydroxy bridges. For example, the average Fe-O bond for the studied structure is 1.961 Å and is within the range of 1.939–2.033 Å reported for related structures. This value is significantly larger than the respective Fe-O_oxo_ bond lengths of 1.804–1.826 Å previously reported for similar cages. Similarly, the Fe^…^Fe distances for the iron atoms bridged by hydroxy ligand (Fe_OH_-Fe_OH_) are longer than the corresponding oxo-mediated Fe^…^Fe distances (Fe_O_^…^Fe_O_). The mean value of 3.744 Å obtained for **I** is even longer than relevant hydroxo mediated Fe^…^Fe distances in structures containing mixed oxo/hydroxo bridges. The studied structure **I** is characterized by a significantly larger Fe-O_OH_-Fe angle than the analogus angles observed in the reference structures. At the same time, the relevant O_OH_-Fe-O_OH_ angle is lower than that reported for the hydroxy bridged fragments of similar structures. These geometrical differences may arise from the fact that the structure with pure hydroxo bridges is not affected by the delocalization of electrons of oxo bridges observed in mixed oxo-hydroxo bridged structures. The delocalization cause elongation of the respective Fe-O_oxo_ bond with simultaneous shortening of the Fe-O_OH_ bonds. The overlay of [Fe_4_O_6_] cores of compared structures is shown on the Figure 5b.

The core overlay diagram for compared structures in Figure 5 revealed that the root mean square deviation (RMSD) values for structures EYAVIJ, JAZZUF and YAXZON range from about 0.08 to 0.09. This strongly indicates that the cores compare well despite the different number of oxo and hydroxo bridging oxygen atoms within these structures. This value increases to about 0.15 for the overlay of mixed oxo/hydroxo cores with the core of **I** containing hydroxo bridges only. This shows that the mean core dimensions are relatively stable for varying the ratio of oxo to hydroxo oxygen atoms and markedly different from the core containing only the OH^−^ bridging units.

### 3.3. Hirshfeld Surface Analysis

The Hirshfeld Surface (HS) and Fingerprint plot (FP) analyses, originally developed for exploration of intermolecular effects governing supramolecular assemblies in crystal structures, are becoming increasingly popular for routine structural investigations [27,28,29,30,31]. These methods are applied for analysis of both intramolecular and intermolecular structural effects in **I**.

#### 3.3.1. Hirshfeld Surface Analysis of Bridging Oxygen Atoms *µ*-OH′ and *µ*-OH″

**Figure 6:**
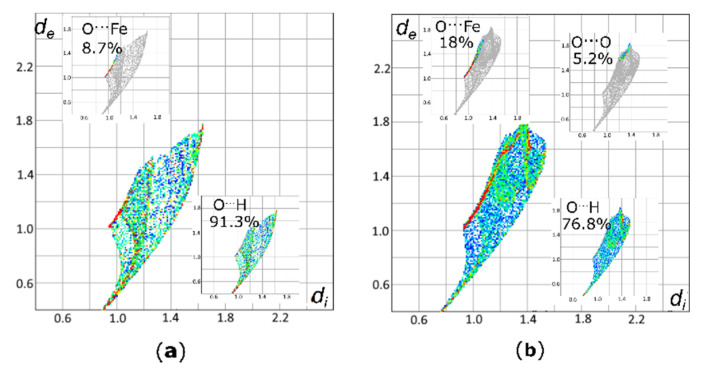
Fingerprint plots (FPs) for the Hirshfeld surface (HS) of (**a**) O1 and (**b**) O2 atoms. The extracted FP for particular contacts are presented together with their respective contributions.

The bridging O-atoms in the [Fe_4_O_6_] adamantane-like framework of **I** consist of six [*µ*-OH]^–^ units. These units are defined by four O1 and two O2 atoms which are symmetrically independent. The O2 atoms are placed ideally on the 4-fold rotonversion axis. At first sight, both oxygen atoms do not differ from each other and form Fe-O bonds characterized by similar parameters. However, fingerprint plots (FP) generated for Hirshfeld surfaces (HS) of symmetrically independent oxygen atoms O1 and O2 revealed differences in their intramolecular environments (Figure 6). The HS of O1 is defined by only two types of intramolecular contacts, O^…^Fe and O^…^H. Contacts with the metal centers, O^…^Fe (8.7%), are represented mainly by green and red dots extending at scattergrams from about *d_e_* = 1 Å to *d_e_* = 1.4 Å and *d_i_* = 0.9 Å to *d_i_* =1.2 Å. Spots representing contacts with hydrogen atoms, O^…^H (91.3%), are more spread. The shortest contact, visible as the second spike and starting from the values of *d_i_* = 0.9 Å and *d_e_* = 0.5 Å, represent the hydrogen atom forming a bond with the O1 oxygen atom. Other scattered spots above these values reflect a contribution of the O^…^H contacts with hydrogen atoms from one CH_3_ group of the *t*-BuOH ligand and the phenyl rings surrounding the core.

A similar FP analysis for O^…^H contacts of the O2 atom demonstrates significant differences and contributions of particular contacts to the HS are different than those observed for the O1 atom. Thus, with increasing contributions from the O^…^Fe contacts (18.0%), diminishing contributions of the scattered spots from the O^…^H interactions (76.8)% are observed. Moreover, a decomposition of the FP for O2 into individual contacts revealed the presence of O^…^O contacts at the level of 1.6%, which are absent for O1. This long-range (*d_i_* ≈ 1.2–1.4 Å and *d_e_* ≈ 1.6–1.8 Å) intramolecular O^…^O contact follows the formation of a homonuclear intramolecular interaction between the O2 and the oxygen atom of the phosphoryl group, O4. The analysis revealed subtle differences between the two bridging oxygen atoms, O1 and O2. Although geometries characterizing the O-Fe bonds are comparable for O1 and O2, the contributions of the respective contacts to their HS differ significantly. Interestingly, the analysis revealed that the O1 atom forming the adamantane-like core is more hindered by hydrophobic groups, such as the CH_3_ fragment of the *t*-BuOH ligand and the CH groups of the phenyl rings, while O2 is more exposed and capable of formation of other contacts. Such distinctions of seemingly equivalent cage-internal atoms are not so striking in the case of classical structural analysis based on geometrical parameters alone. These differences can be reveled and understood only with the HS analysis.

#### 3.3.2. Hirshfeld Surface Analysis for Intermolecular Contacts

**Figure 7:**
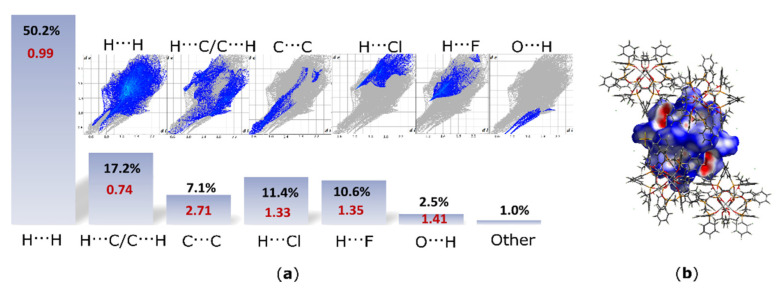
(**a**) 2D fingerprint plots (FPs) for the hexacation of **I** with the percentage contributions to the Hirshfeld surface (HS) areas for the major intermolecular contacts and their respective enrichment ratio (ER)—in red; *d_e_* and *d_i_* are the distances to the nearest atom exterior and interior to the surface, respectively; ER values were not computed for the actual contacts’ contribution below 2% and random contacts below 1%, as they are not meaningful. (**b**) Hirshfeld surfaces (HSs) generated for the cation of **I**; *d_norm_* is visualized with a fixed color scale from −0.655 (red), 0.428 (white) to 1.442 (blue).

For characterization of supramolecular features of the cation in structure **I**, a 3D HS and relevant FP maps were generated (Figure 7). Moreover, the HS and FP analysis has been supported by calculations of the enrichment ratio (ER) [16]. The ER parameter is a valuable tool for quantification of the propensity of individual species to the formation of particular interactions within the crystal lattice. The enrichment ratio (ER_XY_) refers to a pair of elements X and Y forming intermolecular contacts. The ER value allows for a comparison of the actual contacts in the crystal with those computed under the assumption that all contact types have the same probability of formation. If the enrichment ratio is higher than unity, then the given pair of elements is regarded as prone to formation of particular interactions in the crystal. On the other hand, pairs with a low propensity to the formation of contacts with each other have *ER_XY_* < 1. This approach, in contrast to the classical HS and FP analysis, enables to highlight statistically favored contacts having crucial contributions to the crystal packing. A breakdown of the FP into individual contacts showed that the H^…^H contacts comprises 50.2% of the total HS area and are accompanied by the ER value (*ER_HH_* = 0.99) typical for compounds with high hydrogen content on the surface. However, hydrogen atoms, which are involved in electrostatic interactions, such as C-H^…^F, C-H^…^Cl and C-H^…^O (11.4% contact for H^…^Cl and 10.6% contact for H^…^F), are characterized by high and similar ER values, i.e., *ER_HCl_* = 1.33 and *ER_HF_* = 1.35. These values indicate that these electrostatic interactions between the central cation and the surrounding anions are equally engaged in the crystal packing. Although, C-H^…^O hydrogen bonds are represented by a quite low actual contribution (2.5%) of the O^…^H contacts, the value *ER_OH_* = 1.41 indicates the enriched propensity of the species to form these interactions. The C^…^C contacts, usually representing π^…^π interactions, comprise 7.1% of the HS area and are characterized by high ER value (*ER_CC_* = 2.71). Such a high ER value results from a large total area of aromatic rings surrounding the [Fe_4_O_6_] core and their interactions with each other through space in the crystal. Interestingly, although a quite high actual population (17.1%) of the C^…^H contacts, the ER value calculated for this pair (*ER_CH/HC_* = 0.74) suggests that π-facial hydrogen bonds (C^…^H interactions) are disfavored in this structure and are replaced by the π^…^π effects. Moreover, the HS analysis revealed a contribution of C^…^F contacts of only 0.4% accompanied by *ER_CF_* = 0.25. These contacts follow the π^…^F effects, and the propensity to form this kind of interaction is diminished by the preferred H^…^F contacts.

Although geometrical parameters suggested a notable role for the C-H^…^π interactions in stabilization of the crystal lattice, the HS analysis supported by ER calculations revealed that the actual contribution of these interactions is diminished by the π^…^π effects. Indeed, the latter is not formed directly by parallelly assembled aromatic rings and therefore typical bright zones are not observed in the FP analysis. Moreover, the analysis revealed that both PF_6_^−^ and Cl^−^ ions have similar contributions to the structure stabilization. Additionally, the analysis showed that the structure has a higher propensity to form H^…^F contacts than C^…^F contacts following the π^…^F interactions.

## 4. Conclusions

In summary, we reported a comprehensive structural characterization of [(*µ*-OH′)_2_(*µ*-OH″)_4_(*µ*-L)_4_{Fe(*t*-BuOH)}_4_](PF_6_)_2_(Cl)_4_, which contains the [Fe_4_O_6_] heteraadamantane core. This is the first reported structure containing the the Fe/O heteradamantane with a chelating Ph_2_P(O)-CH_2_CH_2_-P(O)Ph_2_ ligand. Moreover, the presented scaffold of the Fe complex is unique and exclusively formed by hydroxy bridging groups, while relevant structures deposited in Cambridge Structural Database contain mixed types of oxygen bridging atoms. The heteraadamantane-type cluster in **I** may be of significant interest due to the strain of the ring systems, which may affect anisotropic magnetic properties of the material. The structure was compared to three other Fe complexes containing the [Fe_4_O_6_] cage with mixed oxo/hydroxo bridging atoms and chelated by the triazacyclononane ligand. The comparison of geometrical parameters showed that the cage is characterized by a notably larger Fe-O_OH_-Fe angle and lower O_OH_-Fe-O_OH_ angle than those reported structures with hydroxy bridged fragments. A purely hydroxo bridged cage of **I** is not affected by the delocalization of electrons of oxo bridges inducing an elongation of the respective Fe-O_oxo_ bonds with shortening of the Fe-O_OH_ bonds. Although the [Fe_4_O_6_] core of the reported structure is almost fully encapsulated by the ligands, the Hirshfeld surface analysis of the bridging oxygen atoms revealed that one of them is more hindered, while the other one is more exposed to interatomic contacts. From the supramolecular point of view, the Hirshfeld surface analysis of the structure revealed that the large total area of aromatic rings surrounding the [Fe_4_O_6_] core leads to π-effects within the crystal lattice. Moreover, enrichment ratio calculations confirmed the high propensity of the title complex to form C-H^…^Cl, C-H^…^F and C-H^…^O interactions.

## Figures and Tables

**Table 1:** Crystal data and structure refinement parameters.

Structure I (CCDC 2109754)	
Formula	C_120_H_142_Cl_4_F_12_Fe_4_O_18_P_10_
D_calc_./ g cm^−3^	1.411
*m*/mm^−1^	6.063
Formula Weight	2775.23
Colour	orange
Size/mm	0.41 × 0.27 × 0.17
T/K	278(2)
Crystal System	Cubic
Flack Parameter	−0.007(4)
Space Group	*I*-43*d*
a = b = c/Å	33.9715(3)
α = β = γ/˚	90
V/ Å^3^	39,205.2(10)
Z/Z’	12/0.25
Wavelength/ Å	1.54184
Radiation type	Cu K*_a_*
*θ*min/°	3.680
*θ*max/°	78.756
Measured Refl’s.	33,119
Independent Refl’s.	5926
Refl’s I ≥ 2 *σ*(I)	4837
*R*_int_	0.042
Parameters	421
Largest Peak	0.34
Deepest Hole	−0.24
GooF	1.035
*wR*_2_(all data)	0.1624
*wR*_2_	0.1514
*R*_1_ (all data)	0.0680
*R*_1_	0.0554

**Table 2:** Geometrical parameters characterizing the selected intramolecular interactions in structure **I**.

**C-H^…^O Interactions**	**C—H/Å**	**H^…^A/Å**	**H^…^A/Å**	**D—H^…^A/˚**
C2—H2^…^O4	0.93	2.48	2.907 (15)	108
C26—H26a^…^O4 ^i^	0.93	2.14	2.962(15)	147
**C-H^…^π Interactions**	**C—H/Å**	**H^…^*Cg*/Å**	**C^…^*Cg*/Å**	**C-H^…^*Cg*/˚**
C16—H16a^…^*Cg*1	0.93	2.87	3.62 (1)	139
C15—H15b^…^*Cg*8 ^ii^	0.93	2.83	3.79(2)	173

Symmetry codes: ^i^ x, 1 − y, ½ − z; ^ii^ 1 − z, 1 ½ − x, y.

**Table 3:** Geometrical parameters characterizing the selected intermolecular interactions in structure **I**.

**C-H^…^F Interactions**	**C—H/Å**	**H^…^A/Å**	**H^…^A/Å**	**D—H^…^A/˚**
C17—H17b^…^F2	0.97	2.46	3.30(1)	145
C6—H6^…^F3 ^i^	0.93	2.63	2.23(1)	124
**F^…^π Interaction**	**-**	**F^…^Cg/Å**	**F^…^Plane/Å**	**-**
F3^…^Cg3	-	4.057	3.165	-

Symmetry code: ^i^ 1.5 − x, y, 1 − z.

**Table 4:** The geometrical parameters characterizing Fe adamantane-like structures containing oxo (O_oxo_) and hydroxo (O_OH_) bridging oxygen atoms. Fe_O_^…^Fe_O_—oxo mediated Fe^…^Fe distance; Fe_OH_^…^Fe_OH_—hydroxo mediated Fe^…^Fe distance; Mean/Min/Max values are given.

Parameter	I	EYAVIJ	JAZZUF	YAXZON
**Bond length/Å**				
Fe-O_OH_	1.961/1.951/1.983	1.986/1.950/2.030	1.996/1.974/2.019	1.996/1.939/2.033
Fe-O_oxo_	-	1.826/*/*	1.816/1.816/1.820	1.806/1.804/1.815
**Angle/°**				
Fe-O_OH_-Fe	145.0/144.5/146.3	128.6/126.8/129.4	124.5/123.7/125.4	124.2/121.1/126.6
Fe-O_oxo_-Fe	-	137.5/*/*	132.6/132.5/132.7	132.3/131.4/133.1
O_OH_-Fe-O_OH_	86.7/85.3/88.5	95.73/89.3/101.9	95.9/93.9/97.6	96.5/92.4/101.5
O_oxo_-Fe-O_oxo_	-	-	-	-
**Distance/Å**				
Fe_OH_^…^Fe_OH_	3.744/3.737/3.747	3.582/3.537/3.624	3.534/3.509/3.564	3.542/3.521/3.555
Fe_O_^…^Fe_O_	-	3.403/*/*	3.325/3.323/3.328	3.331/3.294/3.366

* EYAVIJ contains only one oxo bridging oxygen atom.

## Data Availability

All the data is available within the manuscript, the Supplementary Materials and CCDC: 2109754 which contains the supplementary crystallographic data for this paper. The data are provided free of charge by The Cambridge Crystallographic Data Centre via www.ccdc.cam.ac.uk/structures.

## Supplementary Materials

The following data are available online at https://www.mdpi.com/article/10.3390/ma14226840/s1: Structure solution and refinement details, Figure S1: The IR spectrum of **I** in KBr pellet. Figure S2: The TOF-MS (ES+) spectrum of **I**. Table S1: Structural Data. Table S2: Fractional Atomic Coordinates and Equivalent Isotropic Displacement Parameters for **I**. Table S3: Anisotropic Displacement Parameters for **I**. Table S4: Bond Lengths for **I**. Table S5: Bond Angles for **I**. Table S6: Torsion Angles in for **I**. Table S7: Atomic Occupancies for all atoms that are not fully occupied in **I**. Table S8: Detailed Results of Searches for Mixed Oxo-/Hydroxy Bridged Structures with Adamantane-like Cage.
