# Supplementary material for: Structure of a Fe4O6-Heteraadamantane-Type Hexacation Stabilized by Chelating Organophosphine Oxide Ligands
Source: Materials (Basel). 2021 Nov 12;14(22):6840. doi: 10.3390/ma14226840 (PMC8617765; doi:10.3390/ma14226840)
Supplement: Supplementary file 1 [file materials-14-06840-s001.zip › materials-1430477-supplementary.pdf]

materials

Article

Structure of a Fe_4O_6-Heteraadamantane-Type Hexacation Stabilized by Chelating Organophosphine Oxide Ligands

Anna Pietrzak [1,*], Jannick Guschlbauer [2] and Piotr Kaszyński [2,3,4]

1 Institute of General and Ecological Chemistry, Łódź University of Technology, Żeromskiego 116, 90-924 Łódź, Poland
2 Centre of Molecular and Macromolecular Studies, Polish Academy of Sciences, Sienkiewicza 112, 90-001 Łódź, Poland; J.Guschlbauer@gmx.de (J.G.); piotrk@cbmm.lodz.pl (P.K.)
3 Department of Chemistry, Middle Tennessee State University, Murfreesboro, TN 37132, USA
4 Faculty of Chemistry, University of Łódź, Tamka 12, 91-403 Łódź, Poland
* Correspondence: anna.pietrzak.1@p.lodz.pl

1. Characterization of Compound I

The FT-IR spectrum of **I** was registered on the Jasco FT/IR 6200 spectrophotometer in the spectral range 4000–400 cm^{-1}, with 1 cm^{-1} resolution. Sample was prepared in the form of KBr pellet (sample : KBr ratio was 1:50).

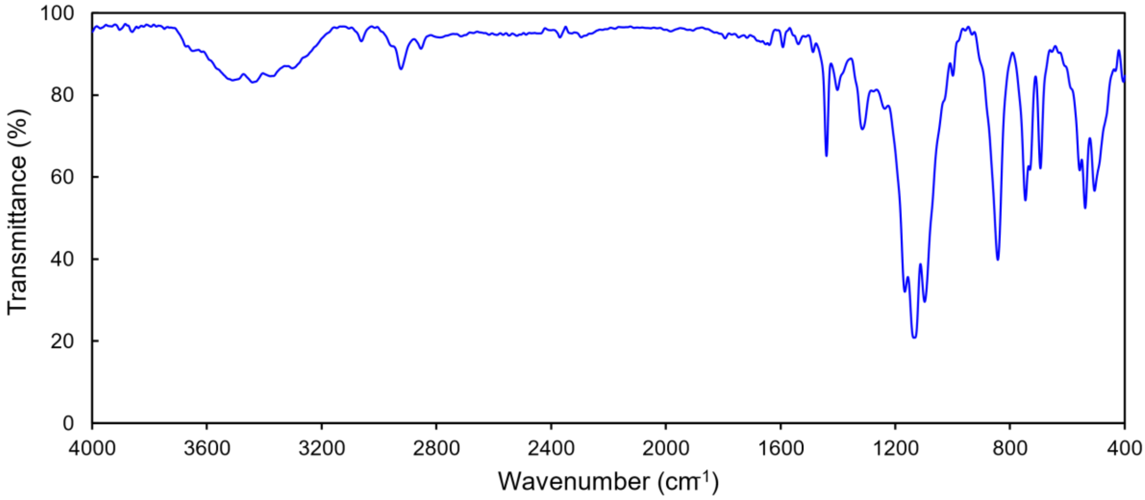

Figure S1. The IR spectrum of **I** in KBr pellet. Resolution 1 cm^{-1}. IR (KBr) *v* 3650-3300 br, 3060 w, 2957 w, 2923 w, 2855 w, 1641 w, 1592 w, 1538 w, 1487 w, 1440 m, 1402 m, 1315 m, 1235 w, 1167 s, 1135 s, 1097 s, 842 s, 746 m, 694 m, 557 m, 538 m, 505 m cm^{-1}.

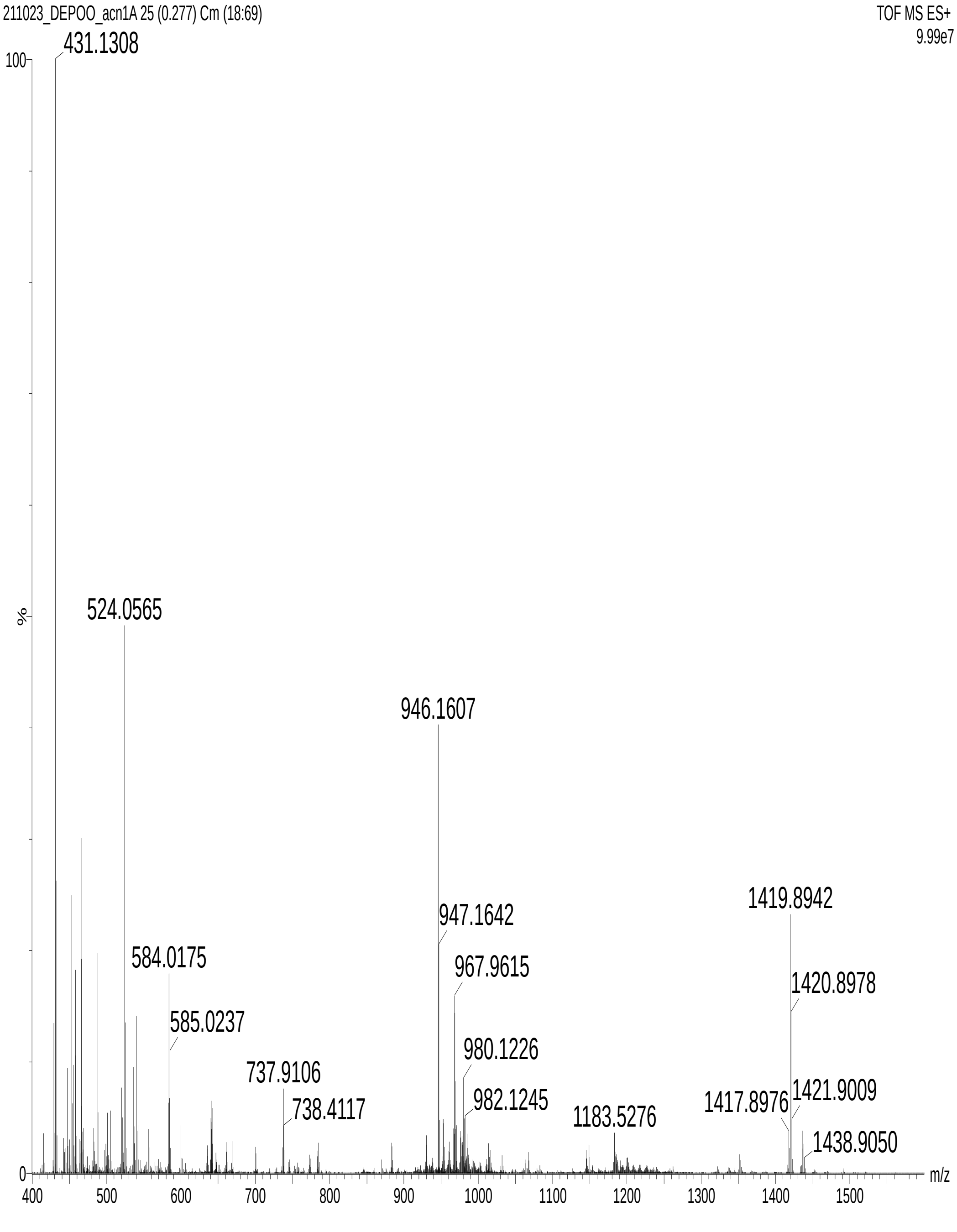

Figure S2. The TOF-MS (ES+) spectrum of **I**.

2. Structure Solution and Refinement Details

An orange block-shaped-shaped crystal with dimensions 0.41 × 0.27 × 0.17 mm^3 was mounted. Data were collected using a XtaLAB Synergy, Dualflex, Pilatus 300K diffractometer operating at T = 278(2) K. Data were measured using ω scans using Cu K_α radiation. The diffraction pattern was indexed and the total number of runs and images was based on the strategy calculation from the program CrysAlisPro software [1]. The maximum resolution that was achieved was $\theta = 78.756°$ (0.79 Å). The unit cell was refined using CrysAlisPro on 13101 reflections, 40% of the observed reflections. Data reduction, scaling and absorption corrections were performed using CrysAlisPro. The final completeness is 99.80 % out to 78.756° in θ. A gaussian absorption correction was performed using CrysAlisPro. Numerical absorption correction based on gaussian integration over a multifaceted crystal

model Empirical absorption correction using spherical harmonics, implemented in SCALE3 ABSPACK scaling algorithm. The structure was solved and the space group *I*-43*d* (# 220) and determined by the ShelXT [2] structure solution program using dual methods and refined by full matrix least squares minimization on F^2 using ShelXL software [3]. All non-hydrogen atoms were refined anisotropically. Hydrogen atom positions were calculated geometrically and refined using the riding model. Most hydrogen atom positions were calculated geometrically and refined using the riding model, but some hydrogen atoms were refined freely. The Flack parameter was refined to -0.007(4). Determination of absolute structure using Bayesian statistics on Bijvoet differences using the Olex2 [4].

Table S1. Structural Data.

	**Structure I**
Formula	$C_{120}H_{142}Cl_4F_{12}Fe_4O_{18}P_{10}$
$D_{calc.}$/ g cm^{-3}	1.411
m/mm^{-1}	6.063
Formula Weight	2775.23
Colour	orange
Shape	block-shaped
Size/mm^3	0.41×0.27×0.17
T/K	278(2)
Crystal System	cubic
Flack Parameter	-0.007(4)
Space Group	*I*-43*d*
a/Å	33.9715(3)
b/Å	33.9715(3)
c/Å	33.9715(3)
a/°	90
b/°	90
g/°	90
V/Å^3	39205.2(10)
Z	12
Z'	0.25
Wavelength/Å	1.54184
Radiation type	Cu K_a
θ_{min}/°	3.680
θ_{max}/°	78.756
Measured Refl's.	33119
Indep't Refl's	5926
Refl's I≥2 s(I)	4837
R_{int}	0.0424
Parameters	421
Restraints	495
Largest Peak	0.337
Deepest Hole	-0.240
GooF	1.035
wR_2 (all data)	0.1624
wR_2	0.1514
R_1 (all data)	0.0680
R_1	1.554

3. Structural Parameters

Table S2. Fractional Atomic Coordinates (×10[4]) and Equivalent Isotropic Displacement Parameters (Å[2]×10[3]) for **I**. U_{eq} is defined as 1/3 of the trace of the orthogonalized U_{ij}.

Atom	x	y	z	U_{eq}
Fe	6641.1(3)	4474.3(3)	2661.4(3)	56.9(3)
P1	7163.3(5)	4505.2(5)	3508.0(5)	63.0(4)
O1	6809.2(16)	5000	2500	56.6(12)
Cl4	5820(50)	7280(40)	6010(40)	950(40)
C1	7642(2)	4732(2)	3514(2)	76.0(16)
P2	6388.9(6)	5477.1(5)	3808.6(5)	71.2(4)
O2	6237.3(10)	4492.4(10)	2252.3(10)	50.4(8)
C2	7781(3)	4888(5)	3178(4)	147(5)
O3	7026.7(16)	4219.5(18)	2291.7(15)	86.9(14)
C3	8155(5)	5051(7)	3163(5)	215(10)
P3	7500	5037.2(15)	5000	125.0(13)
C6	7852(4)	4778(5)	3846(4)	158(6)
C7	7200.6(19)	4017(2)	3713(2)	71.3(15)
C8	7241(3)	3944(3)	4114(3)	95(2)
C9	7290(4)	3569(3)	4244(3)	114(3)
O5	6072.4(15)	5353.0(17)	3531.0(14)	84.7(13)
C5	8224(5)	4948(8)	3846(6)	215(10)
O4	7026.9(12)	4486.7(13)	3090.5(13)	69.6(10)
C4	8376(4)	5069(5)	3484(6)	172(6)
F3	7109(2)	5045(4)	5242(3)	195(3)
C25A	6440(4)	5976(2)	3740(3)	83(4)
C26A	6678(5)	6104(3)	3432(4)	122(7)
C27A	6733(6)	6504(3)	3369(4)	171(13)
C28A	6550(7)	6777(2)	3613(5)	165(13)
C29A	6312(6)	6649(3)	3920(5)	176(13)
C30A	6257(5)	6249(3)	3984(4)	119(7)
F4	7500	5444(5)	5000	296(9)
F2	7260(3)	5031(7)	4638(3)	340(9)
F1	7500	4610(6)	5000	416(15)
C10	7272(4)	3267(3)	3998(4)	122(3)
C11	7237(3)	3330(2)	3594(4)	110(3)
C12	7191(2)	3704(2)	3452(3)	87.7(19)
C13	7397.4(18)	4044(2)	2275(2)	74.6(17)
C14	7461(7)	3684(6)	2465(8)	253(10)
C15	7509(6)	4057(7)	1847(4)	205(8)
C16	7714(4)	4276(7)	2421(7)	202(7)
C17	6824(2)	4783(2)	3807(2)	71.1(15)
C18	6840(2)	5218(2)	3701(2)	75.7(16)
C19	6222(2)	5378(2)	4300(2)	77.4(16)
C20	5885(3)	5182(3)	4354(3)	103(2)
C21	5748(4)	5113(4)	4738(4)	134(4)
C22	5956(5)	5237(4)	5052(3)	136(4)
C23	6303(5)	5437(4)	4999(3)	138(4)
C24	6437(3)	5515(4)	4621(3)	111(3)
C25B	6586(4)	5987(3)	3818(5)	82(4)
C26B	6935(5)	6105(4)	3994(7)	164(14)

Atom	x	y	z	U_{eq}
C27B	7056(4)	6495(5)	3967(7)	152(11)
C28B	6827(5)	6767(3)	3763(7)	142(10)
C29B	6477(5)	6648(4)	3587(7)	175(19)
C30B	6357(4)	6258(4)	3614(6)	132(11)
Cl1	8997(13)	3997(13)	1003(13)	950(40)
Cl3	4311(15)	4311(15)	4311(15)	950(40)

Table S3. Anisotropic Displacement Parameters (×10[4]) for **I**. The anisotropic displacement factor exponent takes the form: $-2p^2[h^2a^{*2} \times U_{11}+ \ldots +2hka^* \times b^* \times U_{12}]$.

Atom	U_{11}	U_{22}	U_{33}	U_{23}	U_{13}	U_{12}
Fe	58.2(5)	55.9(5)	56.6(5)	-2.6(4)	-5.0(4)	6.2(4)
P1	61.5(8)	61.9(8)	65.6(9)	-0.7(7)	-10.6(7)	4.3(7)
O1	48(3)	60(3)	63(3)	13(3)	0	0
C1	67(3)	72(4)	89(4)	-1(3)	-8(3)	-4(3)
P2	91.8(11)	64.7(9)	57.2(8)	-6.7(7)	1.5(8)	15.3(8)
O2	54.8(19)	48.7(17)	47.8(18)	-1.7(15)	-8.7(15)	6.1(15)
C2	100(6)	225(14)	118(7)	43(8)	-13(5)	-61(8)
O3	81(3)	106(4)	74(3)	-2(3)	-7(2)	19(3)
C3	140(11)	330(30)	174(11)	50(16)	-2(9)	-126(14)
P3	120(3)	142(3)	113(3)	0	-37(2)	0
C6	128(8)	231(15)	115(7)	36(9)	-45(6)	-82(9)
C7	63(3)	69(3)	82(4)	6(3)	-6(3)	7(3)
C8	108(6)	92(4)	85(4)	10(4)	-11(4)	5(4)
C9	138(8)	103(5)	102(6)	33(4)	-8(6)	8(6)
O5	90(3)	103(4)	61(2)	-9(2)	-3(2)	24(3)
C5	144(11)	310(20)	192(12)	47(15)	-70(10)	-111(14)
O4	71(2)	71(2)	68(2)	-4.4(19)	-13.0(19)	5(2)
C4	100(8)	205(14)	213(13)	13(12)	-20(7)	-66(9)
F3	138(5)	291(11)	157(6)	-6(7)	-14(4)	-21(6)
C25A	123(13)	67(6)	60(7)	-8(5)	-17(7)	19(6)
C26A	210(20)	72(7)	81(10)	-6(7)	28(10)	4(10)
C27A	330(40)	81(9)	105(14)	6(9)	35(17)	-16(14)
C28A	310(40)	51(8)	132(17)	-14(9)	-10(18)	-14(13)
C29A	310(40)	65(7)	160(20)	-42(10)	28(19)	12(14)
C30A	188(19)	70(7)	98(11)	-19(7)	12(10)	27(9)
F4	340(20)	137(7)	410(30)	0	98(17)	0
F2	176(9)	700(30)	141(6)	-157(11)	-68(6)	123(13)
F1	211(18)	150(7)	890(50)	0	70(20)	0
C10	136(8)	82(5)	148(7)	29(5)	-12(7)	4(5)
C11	132(7)	62(4)	137(6)	4(4)	-7(6)	6(4)
C12	96(5)	70(4)	97(5)	-3(3)	-7(4)	8(4)
C13	57(3)	97(4)	70(4)	7(3)	15(3)	35(3)
C14	242(19)	195(13)	320(20)	123(17)	18(19)	88(12)
C15	192(14)	290(20)	131(8)	-10(9)	76(9)	70(15)
C16	101(7)	276(18)	231(15)	-10(15)	-25(10)	-14(10)
C17	73(4)	71(3)	69(4)	2(3)	-5(3)	8(3)
C18	84(4)	66(3)	77(4)	-8(3)	1(3)	-2(3)
C19	96(4)	77(4)	60(3)	-3(3)	1(3)	24(3)
C20	117(6)	96(6)	96(5)	18(5)	6(5)	4(4)
C21	157(9)	126(8)	118(6)	45(6)	39(6)	20(6)

Atom	U_{11}	U_{22}	U_{33}	U_{23}	U_{13}	U_{12}
C22	192(10)	127(9)	89(5)	31(6)	41(6)	71(7)
C23	210(11)	144(9)	59(4)	-2(5)	-15(6)	39(8)
C24	129(7)	130(7)	73(4)	-15(5)	-8(4)	14(6)
C25B	96(11)	71(6)	77(11)	-9(6)	-1(8)	8(5)
C26B	119(16)	97(11)	280(40)	-5(16)	-71(19)	-2(10)
C27B	111(16)	101(11)	240(30)	-45(15)	-21(16)	-10(9)
C28B	121(16)	84(12)	220(30)	-27(13)	7(16)	-17(9)
C29B	170(30)	83(12)	280(50)	70(20)	-60(30)	-43(15)
C30B	129(16)	81(10)	190(30)	44(14)	-57(17)	-28(10)

Table S4. Bond Lengths in Å for **I.**

Atom	Atom	Length
Fe	O1	1.9534(18)
Fe	O2[1]	1.982(3)
Fe	O2	1.954(3)
Fe	O3	2.011(5)
Fe	O5[2]	1.974(5)
Fe	O4	1.961(4)
P1	C1	1.800(7)
P1	C7	1.804(7)
P1	O4	1.493(5)
P1	C17	1.803(7)
C1	C2	1.345(14)
C1	C6	1.345(13)
P2	O5	1.491(5)
P2	C25A	1.720(8)
P2	C18	1.805(7)
P2	C19	1.794(7)
P2	C25B	1.855(10)
C2	C3	1.387(16)
O3	C13	1.395(7)
C3	C4	1.33(2)
P3	F3[3]	1.561(9)
P3	F3	1.561(9)
P3	F4	1.382(17)
P3	F2[3]	1.474(9)
P3	F2	1.474(9)
P3	F1	1.453(19)
C6	C5	1.391(18)
C7	C8	1.391(11)
C7	C12	1.384(10)
C8	C9	1.361(13)
C9	C10	1.325(16)
C5	C4	1.39(2)
C25A	C26A	1.3900
C25A	C30A	1.3900
C26A	C27A	1.3900

Atom	Atom	Length
C27A	C28A	1.3900
C28A	C29A	1.3900
C29A	C30A	1.3900
C10	C11	1.395(17)
C11	C12	1.368(11)
C13	C14	1.399(17)
C13	C15	1.503(15)
C13	C16	1.423(19)
C17	C18	1.521(10)
C19	C20	1.338(12)
C19	C24	1.393(12)
C20	C21	1.405(15)
C21	C22	1.35(2)
C22	C23	1.37(2)
C23	C24	1.389(16)
C25B	C26B	1.3900
C25B	C30B	1.3900
C26B	C27B	1.3900
C27B	C28B	1.3900
C28B	C29B	1.3900
C29B	C30B	1.3900

[1]5/4-x,1/4+z,3/4-y; [2]5/4-x,3/4-z,-1/4+y; [3]3/2-x,+y,1-z

TableS 5. Bond Angles in ° for **I**.

Atom	Atom	Atom	Angle
O1	Fe	O2	88.67(16)
O1	Fe	O2[1]	85.45(15)
O1	Fe	O3	91.6(2)
O1	Fe	O5[2]	174.3(2)
O1	Fe	O4	89.64(18)
O2	Fe	O2[1]	87.24(15)
O2	Fe	O3	91.52(18)
O2[1]	Fe	O3	176.8(2)
O2	Fe	O5[2]	90.23(18)
O2	Fe	O4	175.97(18)
O5[2]	Fe	O2[1]	88.9(2)
O5[2]	Fe	O3	94.1(3)
O4	Fe	O2[1]	88.98(17)
O4	Fe	O3	92.2(2)
O4	Fe	O5[2]	91.1(2)
C1	P1	C7	109.0(3)
C1	P1	C17	110.3(3)
O4	P1	C1	108.0(3)
O4	P1	C7	110.5(3)
O4	P1	C17	111.0(3)
C17	P1	C7	108.0(3)
Fe[3]	O1	Fe	146.0(3)
C2	C1	P1	118.5(7)
C2	C1	C6	118.8(9)

Atom	Atom	Atom	Angle
C6	C1	P1	122.5(8)
O5	P2	C25A	105.4(5)
O5	P2	C18	110.2(3)
O5	P2	C19	107.9(4)
O5	P2	C25B	122.3(5)
C25A	P2	C18	111.5(5)
C25A	P2	C19	110.1(4)
C18	P2	C25B	98.8(6)
C19	P2	C18	111.5(3)
C19	P2	C25B	105.8(6)
Fe	O2	Fe[2]	144.4(2)
C1	C2	C3	120.7(12)
C13	O3	Fe	143.0(4)
C4	C3	C2	120.5(15)
F3	P3	F3[4]	178.0(10)
F4	P3	F3[4]	89.0(5)
F4	P3	F3	89.0(5)
F4	P3	F2[4]	90.9(10)
F4	P3	F2	90.9(10)
F4	P3	F1	180.0
F2[4]	P3	F3	91.8(6)
F2	P3	F3	88.2(6)
F2	P3	F3[4]	91.8(6)
F2[4]	P3	F3[4]	88.2(6)
F2[4]	P3	F2	178.3(19)
F1	P3	F3[4]	91.0(5)
F1	P3	F3	91.0(5)
F1	P3	F2	89.1(10)
F1	P3	F2[4]	89.1(10)
C1	C6	C5	121.9(13)
C8	C7	P1	123.2(6)
C12	C7	P1	117.1(6)
C12	C7	C8	119.6(7)
C9	C8	C7	119.9(9)
C10	C9	C8	120.8(10)
P2	O5	Fe[1]	155.3(3)
C6	C5	C4	117.5(13)
P1	O4	Fe	156.1(3)
C3	C4	C5	120.0(12)
C26A	C25A	P2	118.0(7)
C26A	C25A	C30A	120.0
C30A	C25A	P2	122.0(7)
C25A	C26A	C27A	120.0
C28A	C27A	C26A	120.0
C27A	C28A	C29A	120.0
C30A	C29A	C28A	120.0
C29A	C30A	C25A	120.0
C9	C10	C11	120.5(9)
C12	C11	C10	120.0(9)
C11	C12	C7	118.9(8)

Atom	Atom	Atom	Angle
O3	C13	C14	119.6(12)
O3	C13	C15	104.7(8)
O3	C13	C16	115.5(10)
C14	C13	C15	115.7(14)
C14	C13	C16	101.9(15)
C16	C13	C15	97.5(13)
C18	C17	P1	110.7(5)
C17	C18	P2	113.3(5)
C20	C19	P2	119.5(7)
C20	C19	C24	120.5(9)
C24	C19	P2	120.0(7)
C19	C20	C21	119.7(11)
C22	C21	C20	120.6(13)
C21	C22	C23	120.1(10)
C22	C23	C24	119.9(12)
C23	C24	C19	119.2(12)
C26B	C25B	P2	125.8(8)
C26B	C25B	C30B	120.0
C30B	C25B	P2	114.1(8)
C25B	C26B	C27B	120.0
C28B	C27B	C26B	120.0
C29B	C28B	C27B	120.0
C28B	C29B	C30B	120.0
C29B	C30B	C25B	120.0

[1]5/4-x,1/4+z,3/4-y; [2]5/4-x,3/4-z,-1/4+y; [3]+x,1-y,1/2-z; [4]3/2-x,+y,1-z

Table S6. Torsion Angles in ° for **I**.

Atom	Atom	Atom	Atom	Angle
Fe	O3	C13	C14	66.8(18)
Fe	O3	C13	C15	-161.5(11)
Fe	O3	C13	C16	-55.5(14)
P1	C1	C2	C3	-177.4(16)
P1	C1	C6	C5	178.9(17)
P1	C7	C8	C9	-176.2(8)
P1	C7	C12	C11	177.0(7)
P1	C17	C18	P2	154.1(4)
C1	P1	C7	C8	75.1(7)
C1	P1	C7	C12	-104.3(6)
C1	P1	O4	Fe	-151.7(7)
C1	P1	C17	C18	49.7(6)
C1	C2	C3	C4	-2(3)
C1	C6	C5	C4	0(3)
P2	C25A	C26A	C27A	-179.1(12)
P2	C25A	C30A	C29A	179.1(12)
P2	C19	C20	C21	-178.3(8)
P2	C19	C24	C23	179.8(8)
P2	C25B	C26B	C27B	177.7(14)
P2	C25B	C30B	C29B	-177.9(13)
C2	C1	C6	C5	-6(3)

Atom	Atom	Atom	Atom	Angle
C2	C3	C4	C5	-4(4)
C6	C1	C2	C3	8(2)
C6	C5	C4	C3	6(4)
C7	P1	C1	C2	127.9(10)
C7	P1	C1	C6	-57.5(12)
C7	P1	O4	Fe	89.2(8)
C7	P1	C17	C18	168.8(5)
C7	C8	C9	C10	-4.9(17)
C8	C7	C12	C11	-2.5(13)
C8	C9	C10	C11	6(2)
C9	C10	C11	C12	-5.1(19)
O5	P2	C25A	C26A	-83.8(9)
O5	P2	C25A	C30A	97.2(10)
O5	P2	C18	C17	-70.6(6)
O5	P2	C19	C20	7.1(7)
O5	P2	C19	C24	-171.3(7)
O5	P2	C25B	C26B	-163.7(11)
O5	P2	C25B	C30B	14.1(14)
O4	P1	C1	C2	7.9(10)
O4	P1	C1	C6	-177.5(11)
O4	P1	C7	C8	-166.4(6)
O4	P1	C7	C12	14.2(7)
O4	P1	C17	C18	-69.9(6)
C25A	P2	O5	Fe[1]	90.3(10)
C25A	P2	C18	C17	172.7(6)
C25A	P2	C19	C20	121.6(8)
C25A	P2	C19	C24	-56.7(9)
C25A	C26A	C27A	C28A	0.0
C26A	C25A	C30A	C29A	0.0
C26A	C27A	C28A	C29A	0.0
C27A	C28A	C29A	C30A	0.0
C28A	C29A	C30A	C25A	0.0
C30A	C25A	C26A	C27A	0.0
C10	C11	C12	C7	3.4(16)
C12	C7	C8	C9	3.1(13)
C17	P1	C1	C2	-113.6(10)
C17	P1	C1	C6	61.0(12)
C17	P1	C7	C8	-44.8(8)
C17	P1	C7	C12	135.8(6)
C17	P1	O4	Fe	-30.7(8)
C18	P2	O5	Fe[1]	-30.1(10)
C18	P2	C25A	C26A	35.9(10)
C18	P2	C25A	C30A	-143.2(9)
C18	P2	C19	C20	-114.1(7)
C18	P2	C19	C24	67.6(8)
C18	P2	C25B	C26B	-43.0(13)
C18	P2	C25B	C30B	134.8(11)
C19	P2	O5	Fe[1]	-152.1(9)
C19	P2	C25A	C26A	160.1(8)
C19	P2	C25A	C30A	-19.0(11)

Atom	Atom	Atom	Atom	Angle
C19	P2	C18	C17	49.2(6)
C19	P2	C25B	C26B	72.4(13)
C19	P2	C25B	C30B	-109.8(11)
C19	C20	C21	C22	-1.2(16)
C20	C19	C24	C23	1.5(15)
C20	C21	C22	C23	0.8(18)
C21	C22	C23	C24	0.7(19)
C22	C23	C24	C19	-1.9(17)
C24	C19	C20	C21	0.0(14)
C25B	P2	O5	Fe[1]	85.0(11)
C25B	P2	C18	C17	160.2(7)
C25B	P2	C19	C20	139.5(8)
C25B	P2	C19	C24	-38.8(9)
C25B	C26B	C27B	C28B	0.0
C26B	C25B	C30B	C29B	0.0
C26B	C27B	C28B	C29B	0.0
C27B	C28B	C29B	C30B	0.0
C28B	C29B	C30B	C25B	0.0
C30B	C25B	C26B	C27B	0.0

[1]5/4-x,1/4+z,3/4-y

Table S7. Atomic Occupancies for all atoms that are not fully occupied in **I**.

Atom	Occupancy
H1	0.5
Cl4	0.3333
C25A	0.535(13)
C26A	0.535(13)
H26A	0.535(13)
C27A	0.535(13)
H27A	0.535(13)
C28A	0.535(13)
H28A	0.535(13)
C29A	0.535(13)
H29A	0.535(13)
C30A	0.535(13)
H30A	0.535(13)
C25B	0.465(13)
C26B	0.465(13)
H26B	0.465(13)
C27B	0.465(13)
H27B	0.465(13)
C28B	0.465(13)
H28B	0.465(13)
C29B	0.465(13)
H29B	0.465(13)
C30B	0.465(13)
H30B	0.465(13)

4. CSD Search Details

Table S8. Detailed results of searches for mixed oxo-/hydroxy bridged structures with adamantane-like cage.

Metal at the bridgehead position	Number of oxo-bridging oxygen atoms	Number of hydroxy bridging oxygen atoms	Number of structures deposited in CSD
Any Metal	6	0	45
	5	1	2
	4	2	2
	3	3	1
	2	4	4
	1	5	1
	0	6	6
Any Transition Metal	6	0	36
	5	1	2
	4	2	0
	3	3	0
	2	4	3
	1	5	1
	0	6	3
Fe	6	0	0
	5	1	0
	4	2	0
	3	3	0
	2	4	2
	1	5	1
	0	6	0

References

1. Agilent (2014). CrysAlis PRO. Agilent Technologies Ltd, Yarnton, Oxfordshire, England.
2. Sheldrick, G.M. SHELXT—Integrated space-group and cystal-structure determination. *Acta Cryst. A* **2015**, *71*, 3–8.
3. Sheldrick, G.M. Crystal structure refinement withSHELXL. *Acta Crystallogr. Sect. C Struct. Chem.* **2015**, *71*, 3–8, doi:10.1107/s2053229614024218.
4. Dolomanov, O.V.; Bourhis, L.J.; Gildea, R.J.; Howard, J.A.K.; Puschmann, H. OLEX2: A complete structure solution, refinement and analysis program. *J. Appl. Crystallogr.* **2009**, *42*, 339–341, https://doi.org/10.1107/s0021889808042726.
